# Changes in cardiac structure and function in a modified rat model of myocardial hypertrophy

**DOI:** 10.5830/CVJA-2015-053

**Published:** 2016

**Authors:** Wenjun Dai, Shiming Liu, Qi Dong, Minsheng Chen, Wenjun Dai, Shiming Liu, Luning Zhao, Ailan Chen, Zhenci Li

**Affiliations:** Department of Cardiology, The Second Affiliated Hospital of Guangzhou Medical University, Guangzhou, China; Department of Cardiology, The Second Affiliated Hospital of Guangzhou Medical University, Guangzhou, China; Department of Physiology, Guangzhou Medical University, Guangzhou, China; Guangzhou Institute of Cardiovascular Disease, Guangzhou, China; Guangzhou Institute of Cardiovascular Disease, Guangzhou, China; Guangzhou Institute of Cardiovascular Disease, Guangzhou, China; Department of Medical Experimental Centre, Guangzhou Medical University, Guangzhou, China; Department of Cardiology, The First Affiliated Hospital of Guangzhou Medical University, Guangzhou, China; Department of Cardiology, The First Municipal People’s Hospital of Guangzhou affiliated to Guangzhou Medical College, Guangzhou, China

**Keywords:** AAC, myocardial hypertrophy, echocardiography, rat

## Abstract

**Aim:**

In this study we designed a modified method of abdominal aortic constriction (AAC) in order to establish a stable animal model of left ventricular hypertrophy (LVH). We also evaluated cardiac structure and function in rats with myocardial hypertrophy using echocardiography, and provide a theory and experimental basis for the application of drug interventions using the LVH animal model. We hope this model will provide insight into novel clinical therapies for LVH.

**Methods:**

The abdominal aorta of male Wistar rats (80–100 g) was constricted between the branches of the coeliac and anterior mesenteric arteries, to a diameter of 0.55 mm. Echocardiography, using a linear phase array probe, combined with histology and plasma BNP concentration, was performed at three, four and six weeks post AAC.

**Results:**

The acute (24-hour) mortality rate was lower (8%) than in previous reports (15%) using this modified rat model. Compared with shams, animals who underwent AAC demonstrated significantly increased interventricular septal (IVS), LV posterior wall (LVPWd), LV mass index (LVMI), crosssectional area (CSA) of myocytes, and perivascular fibrosis; while the ejection fraction (EF), fractional shortening (FS) and cardiac output (CO) were consistently lower at each time interval. Notably, differences in these parameters between the AAC and sham groups were significant by three weeks and reached a peak at four weeks. Following AAC, plasma B-type natriuretic peptide (BNP) level was gradually elevated, compared with the sham group, between three and six weeks.

**Conclusion:**

This modified AAC model induced LVH both stably and safely by week four post surgery. Echocardiography was accurately able to assess changes in chamber dimensions and systolic properties in the rats with LVH.

## Aim

The development of left ventricular hypertrophy (LVH) is an adaptive response to pressure overload. Clinically, sustained hypertrophy is correlated with an increase in incidence of cardiovascular disease (CVD)-mediated mortality, and is often the initial step in the progression to congestive heart failure. Cardiac hypertrophy is also a risk factor for arrhythmia and sudden cardiac death. In order to develop therapeutic approaches to prevent LVH, it is important to elucidate the precise mechanisms and time course of the development of LVH.

Animal models of hypertrophy are critically important for studies on the pathogenesis of LVH, its pathological processes and therapeutic strategies for treatment and/or prevention of LVH. Abdominal aortic constriction (AAC) is the most widely used strategy to induce pressure overload leading to compensatory myocardial hypertrophy.

Several important factors may affect the time course and progression of myocardial hypertrophy caused by pressure overload, such as species and age of animals, anatomical sites of constriction, overload duration, and the degree of stenosis.^1^,^2^ In previous studies, most investigators constricted the abdominal aorta above the left renal artery in adult rats. Based on the anatomy of the abdominal aorta in rats, there are two large arterial branches anterior to the left renal artery, the coeliac and anterior mesenteric arteries. The constriction site relative to these arterial branches may affect progression to myocardial hypertrophy.^3^-^6^ Recent reports have noted that younger animals fare better than adults following the AAC procedure,^2^ since younger animals may have a narrower abdominal aorta, leading to suboptimal restriction and less myocardial hypertrophy and AAC-mediated surgical mortality.

In this study, we modified the traditional methods of AAC to evaluate banding severity, banding location, age and time course. We also explored an optimal method to induce myocardial hypertrophy in rats. To date, most studies have used small-animal echocardiographic systems or clinical echocardiography instruments coupled with miniature transducers to detect ventricular structural and functional changes in rats. This specialised equipment increases the cost of experiments.^2^-^4^,^7^ In order to reduce these costs, we evaluated the possibility of using a standard human ultrasound probe, which is commonly found in hospitals, to investigate cardiac changes in sham rats or rats with ACC-induced myocardial hypertrophy by using an extensive analysis, including echocardiography, heart and left ventricular mass, cardiomyocyte area, plasma B-type natriuretic peptide (BNP) concentration, and interstitial and perivascular fibrosis.

BNP is a circulating hormone produced in the heart, primarily by ventricular cells. This hormone is mainly secreted in response to increases in ventricular wall stress, such as ventricular hypertrophy, and it is detectable at high concentrations in a number of circumstances, including cardiac ischaemia and severe heart failure.^8^ In addition, the expression of BNP is significantly increased in animal models of chronic haemodynamic overload. Therefore, we measured blood BNP concentrations to enhance our understanding of BNP secretion in both AAC and sham rats sequentially, as a marker to evaluate the extent of cardiac hypertrophy.

The primary goals of this study were to modify the AAC procedure in order to establish a safer and more stable LVH model in rodents, evaluate the utility of standard human ultrasound probes to detect structural and functional changes in rats with cardiac hypertrophy, and provide a theoretical and experimental foundation for the application of novel drug interventions aimed at interfering with clinical LVH.

## Methods

Fifty male Wistar rats (80–100 g) were used in all experiments. They were allowed standard laboratory chow and tap water *ad libitum* and housed in stable conditions at 22°C with a 12-hour light/dark cycle for one week prior to AAC surgery. All procedures were performed in accordance with institutional guidelines for animal research.

After a one-week acclimatisation, the AAC surgery was performed. All experimental rats were weighed prior to surgery, and at three, four and six weeks post surgery. Echocardiographic studies were conducted at three, four and six weeks post surgery. For LV weight and histological measurements, rats were sacrificed following echocardiography, and blood samples were collected from the right carotid artery for enzyme-linked immunosorbent assay (ELISA) analyses of plasma BNP concentrations.

AAC was induced in outbred male rats, as previously described.^9^ In brief, the animals were anesthetised with sodium pentobarbital (45 mg/kg, i.p.). The abdominal aorta proximal to the left renal artery was exposed and separated from the vena cava. A 2-0 silk suture was tied, using a blunt 24-G probe (the external diameter was 0.55 mm), beside the aorta between the branches of the coeliac and anterior mesenteric arteries. The probe was removed, leaving the vessel constricted to a diameter of 0.55 mm. Saline (1 ml) was administered into the peritoneal cavity in order to replenish any fluid loss, and the abdominal wall and skin were sutured closed. All rats were allowed to recover on a warming pad. The same procedure was performed in the sham animals except that the silk suture around the aorta was pulled through and not tied.

Following surgery, 50 mg/kg, i.m. ampicillin was administered once daily for three days after surgery to prevent infection. The day following surgery, the sham and AAC animals were randomly divided into six groups as follows: (1) sham for three weeks (*n* = 8); (2) sham for four weeks (*n* = 8); (3) sham for six weeks (*n* = 8); (4) AAC for three weeks (*n* = 8); (5) AAC for four weeks (*n* = 8); (6) AAC for six weeks (*n* = 10).

## Echocardiographic studies

Echocardiographic studies were performed between 16:00 and 20:00, with the animals in the left lateral decubitus position, using sodium pentobarbital (45 mg/kg, i.p.) for anaesthesia. The IE 33 echocardiographic system (Philips Medical Systems, Nederland BV) was used to perform two-dimensional (2D) guided M-mode echocardiography and pulse-wave Doppler echocardiography with a linear-phase array probe (L15-7io; frequency range 7–15 MHz), which was placed on the shaved left hemithorax. 2D images of the heart were obtained in the parasternal long-axis view, followed by the short-axis and apical four-chamber views.

M-mode echocardiography is useful for assessing LVH, allowing accurate measurements of wall thickness and LV dimensions during systole and diastole. For M-mode recordings, the parasternal long-axis view was used to image the heart in 2D, with a depth setting of 2 cm. M-mode recordings were then analysed at a sweep speed of 66 mm/s, with the axis of the probe aligned near the posterior leaf mitral valve.

The following parameters were measured: LV posterior wall (LVPW) dimensions during both diastole and systole (LVPWd and LVPWs, respectively), interventricular septal (IVS) dimensions during both diastole and systole (IVSd and IVSs, respectively), LV internal dimensions (LVID) during both diastole and systole (LVIDd and LVIDs, respectively), LV end-diastolic volume (EDV), LV end-systolic volume (ESV), percentage LV fractional shortening (FS), LV ejection fraction (EF), cardiac output (CO), and heart rate (HR). LV mass (LVm) was obtained from echocardiography and derived from the cubic equation at the end of diastole:

LVm = 1.04 × [(LVIDd + LVPWd + IVSd)^3^ – LVIDd^3^] × 0.8 + 0.14.^10^

All data are means of three consecutive cardiac cycles. 2D guided M-mode recordings were obtained from the parasternal short-axis view of the left ventricle at the level of the papillary muscles. The angle of the M-mode beam was focused on the middle of the LV, and aligned at the anteroposterior axis, perpendicular to the LV walls. Parameters and methods were the same as in the parasternal long-axis view, except that the thickness of the LV anterior wall (LVAW) was obtained instead of IVS. To assess inflow and outflow of the left ventricle, Doppler recordings were acquired in the apical four-chamber view to obtain inflow values parallel to the sample volume.

Pulse-wave Doppler (PWD) recordings were acquired with the sample volume placed midway between the mitral and aortic valves to determine the velocity of mitral inflow and aortic outflow. PWD spectra of mitral inflow were recorded with the sample volume placed at the tips of the mitral valve leaflets and adjusted to the position at which velocity was maximal, with the sample volume set to the smallest size available (1 mm). Due to the high heart rates of rodents, which caused fusion of the E and A waves, diastolic function was not evaluated using Doppler imaging.

LV outflow velocity was recorded from the apical long-axis view, with the sample volume positioned just below the aortic valve. The Doppler beam was set within 30° of the incident angle, to the aortic direction. Values for statistical analyses were averaged data collected from three to five cardiac cycles.

Colour-flow Doppler images were obtained by centering the sampling area in the region of interest, thus making it possible to evaluate valvular dysfunction. The same method was used to measure diameters of the abdominal aorta via the aortic longaxis view.

All measurements were performed in accordance with the leading-edge method of the American Society of Echocardiography.^11^ Images were stored digitally on magnetooptical discs (DICOM).

## Morphological analyses

Rat hearts were harvested by cardiectomy and perfused in 30 ml of ice-cold phosphate-buffered saline to wash out any remaining blood. The total mass of the hearts and mass of the right and left ventricles were obtained. Heart mass index (HMI) and LV mass index (LVMI) were determined as the ratio of LVm (in mg) to body weight (in g).

The left ventricle was then cut into four pieces along the longitudinal axis. Three sections were immediately frozen in liquid nitrogen and one was fixed in 10% formalin for histological analysis. Tissues were dehydrated through serial alcohol immersions, cleared in xylene and embedded in paraffin. They were then cut into three to five 5-μm-thick sections for each animal. For cardiomyocyte size measurements, the samples were stained with haematoxylin/eosin (HE) and Masson trichrome (MT), as previously described,^12^ for the evaluation of interstitial and perivascular fibrosis.

To quantify cardiomyocyte hypertrophy, about 30 myocytes were selected randomly per section, at 400 × magnification, and digitally imaged. The cross-sectional area of the cardiomyocytes was measured using Image Pro Plus software (Media Cybernetics, Carlsbad, CA). Quantitative measurement of the perivascular fibrosis area was calculated as the ratio of the fibrosis area surrounding the vessel wall to the total vessel area using Image Pro Plus software. At least 10 arterial cross sections were examined per heart. The area of interstitial fibrosis was identified, after excluding the vessel area from the region of interest, as the ratio of interstitial fibrosis to the total tissue area. At least three sections were examined per heart.

For ELISA analyses, blood was collected into tubes containing heparin from the right carotid artery of the rats and immediately centrifuged at 1 000 × g for 15 minutes. The plasma supernatant was collected and maintained at −80°C for BNP concentration determinations, using ELISA assays with a specific BNP kit (Cusabio).

## Statistical analyses

All data are expressed as mean ± SD. One-way analysis of variance (ANOVA) was performed to compare the AAC and sham groups. When the probability value was statistically significant, a least-significant-difference (LSD) *t*-test was applied for multiple comparisons. Linear regression analysis was used to evaluate correlations between LVm by echocardiography and the actual weights from sacrificed rats. SPSS V19 was used for statistical analyses. A probability value of *p* < 0.05 was considered statistically significant.

## Results

Fifty rats were initially enrolled in this study. After the surgery (same day as surgery), the mortality rate for the experimental pressure overload was 8% (two rats died), and there were no fatalities in the sham groups. Three days after surgery, 41 rats remained in the study, including 22 sham controls and 19 AAC rats. No additional fatalities occurred over the duration of the experiment.

**Fig. 1.**
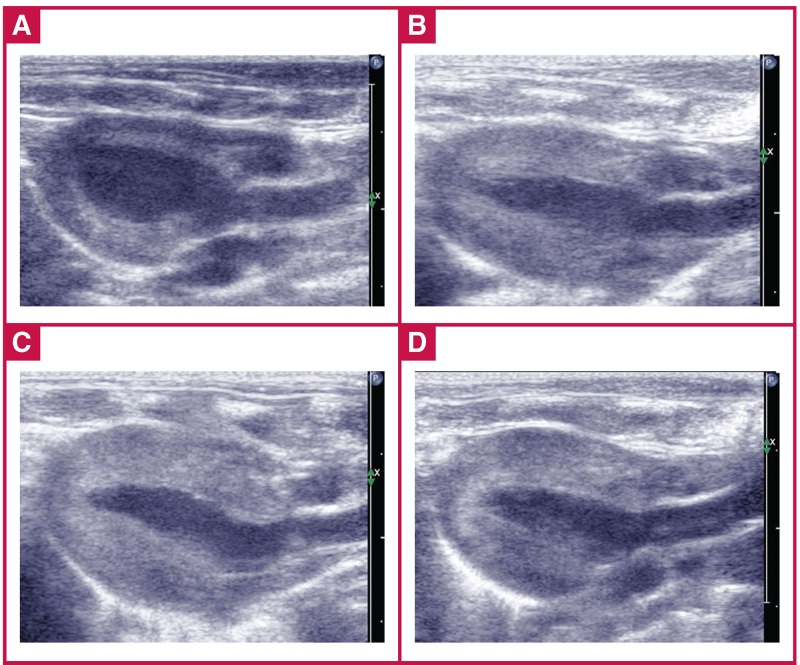
Abdominal aortic constriction. A: sham rats, B: AAC rats, C: sham rats using colour Doppler, D: AAC rats using colour Doppler.

**Fig. 2.**
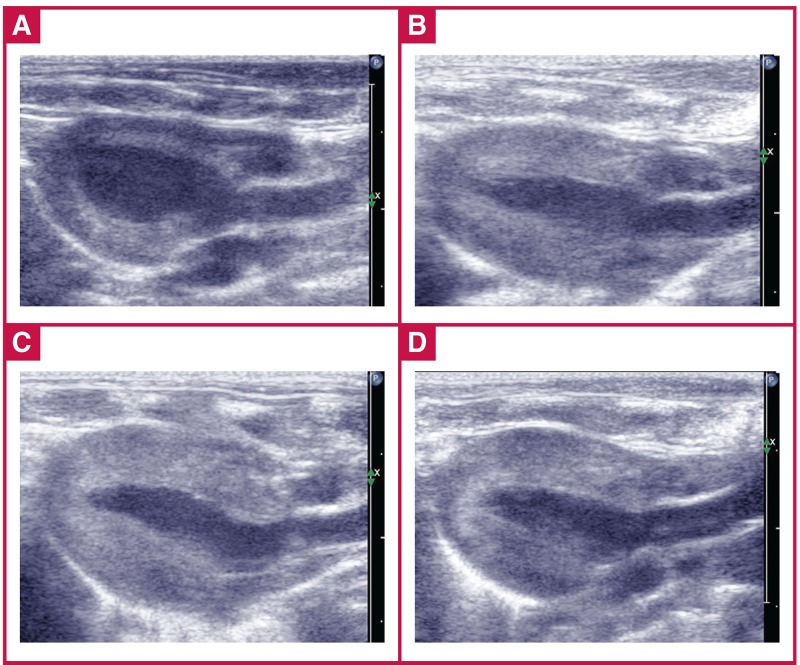
Parasternal long-axis view of the left ventricle. A: sham rats at six weeks, B: AAC rats at three weeks, C: AAC rats at four weeks, D: AAC rats at six weeks..

Following AAC surgery, the abdominal aortas were constricted in diameter by 37% (~ 0.06 ± 0.01 cm) relative to the aorta, measured using echocardiography (Fig. 1). Fig. 2 shows a typical 2D echocardiogram of the heart obtained via the parasternal long-axis view of the LV to compare cardiac structures between the sham and AAC groups at three, four and six weeks.

**Fig. 3.**
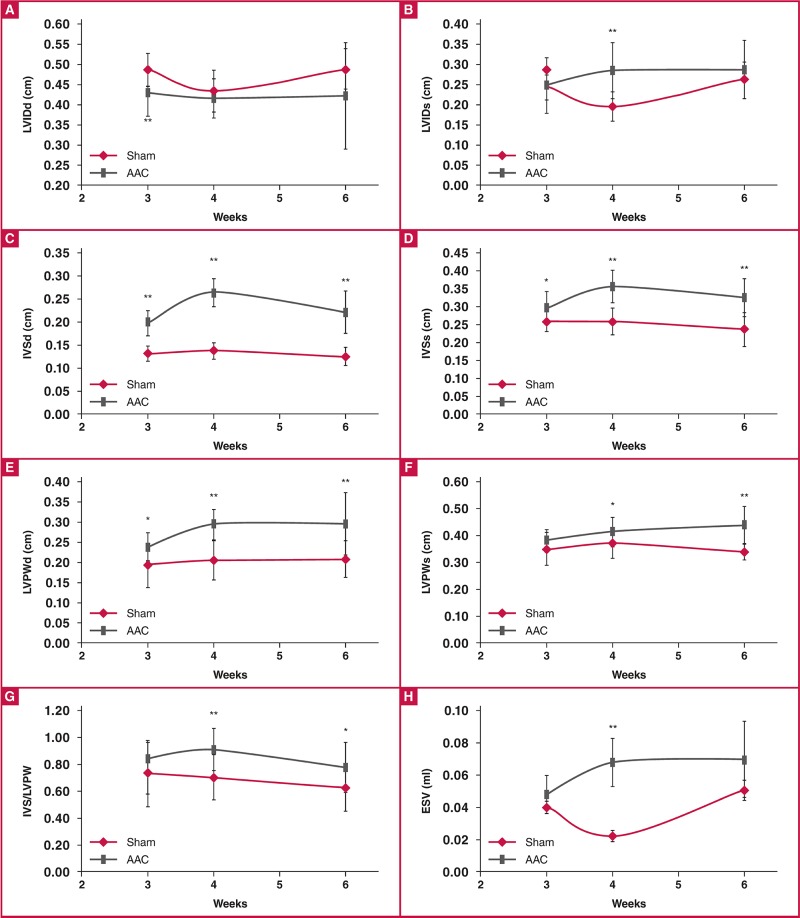
M-mode echocardiographic examination of diastolic dimensions (A, C, E, G) and systolic dimensions (B, D, F, H) in sham and AAC rats; *p < 0.05 vs sham control, **p < 0.01 vs sham control. The n values are given for each group at three, four and six weeks in sequential order. ANOVA was used to compare the AAC and sham groups, when the probability value was statistically significant. An LSD t-test was applied for multiple comparisons.

Fig. 3 demonstrates the M-mode changes that occurred in the LVID, IVS and LVPW dimensions during both diastole (Fig. 3A, C, E) and systole (Fig. 3B, D, F), as well as in EDV and ESV (Fig. 3G, H). IVS (Fig. 3C, D) was significantly increased in AAC rats as early as three weeks post surgery, compared to the controls. This trend continued to four weeks, when the AAC rats exhibited wall thickening of 193% at diastole (137% at systole) compared to the sham values. In addition, the AAC rats exhibited significant thickening of LVPWd at three weeks, which progressed to 143% of the control values at four weeks.

In the AAC rats, IVS and LVPWd were significantly increased at all time points, reaching a maximum at four weeks. At six weeks post AAC, IVSd and LVPWd were decreased to 177% and 141% of the sham values, respectively. IVSs remained at the same level at four and six weeks post AAC, which was 137% of the control values. In the AAC rats, LVPWs were significantly increased (112–129% of the sham values) four weeks post surgery, a trend that continued to six weeks. Subsequently, the AAC rats had no marked chamber dilatation; however, some time points showed statistically significant yet marginal increases in ESV and IVIDs (at four weeks), and EDV and LVIDd were decreased significantly at three weeks post AAC.

**Fig. 4.**
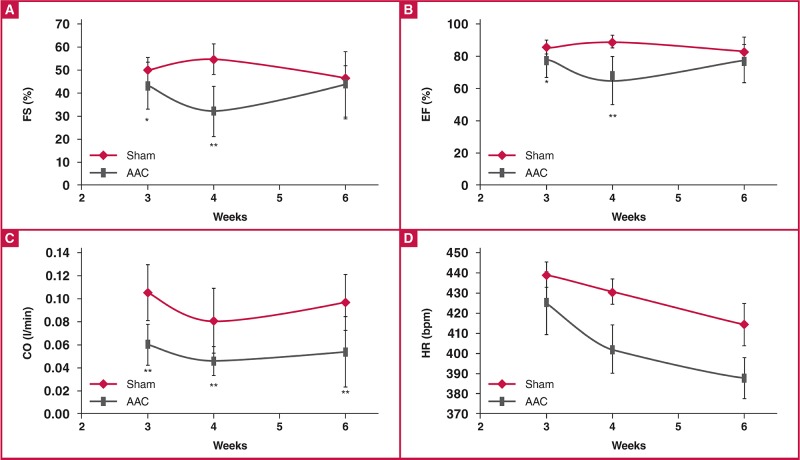
FS (A), EF (B), CO (C) and HR (D) of AAC models. *p < 0.05 vs sham control, **p < 0.01 vs sham control. The n values are given for each group at three, four and six weeks in sequential order. ANOVA was performed to compare the AAC and sham groups when the probability value was statistically significant. An LSD t-test was applied for multiple comparisons.

Fig. 4 shows the changes in FS, EF, CO and HR that occurred over the six-week time course in both sham and AAC rats. In the AAC rats, reduced systolic function was first noted at three weeks post surgery, with significant reductions in FS and EF (Fig. 4A, B). Both of these trends continued to four weeks, where these parameters in the AAC group were markedly decreased (FS: 54.9 ± 6.5% for sham vs 32.3 ± 10.9% for AAC; EF: 89.4 ± 4.0% for sham vs 65.3 ± 15.2% for AAC). FS and EF had slightly recovered at six weeks, but were still decreased compared with the sham values (FS: 46.6 ± 5.0% for sham vs 43.7 ± 14.5% for AAC; EF: 83.0 ± 4.8% for sham vs 77.9 ± 14.2% for AAC). These differences did not reach statistical significance.

CO was measured in order to differentiate between highand low-output failure (Fig. 4C). In the AAC rats, CO was significantly decreased at the three-week time point, and was less than half (55% of sham values) by six weeks. HR was not significantly different between the two groups (Fig. 4D).

**Fig. 5.**
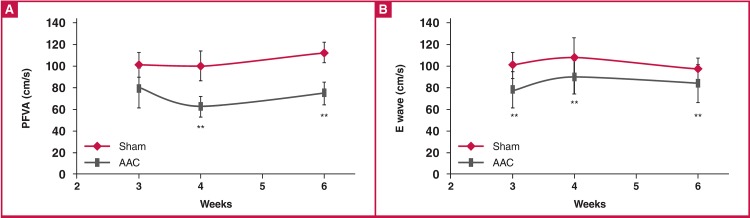
Time course of velocity parameters obtained using Doppler echocardiography in both sham and AAC rats. PFVA, peak flow velocity of aorta; E wave, peak early diastolic filling velocity. *p < 0.05 vs sham control, **p < 0.01 vs sham control. The n values are given for each group at three, four and six weeks in sequential order. ANOVA was performed to compare the AAC and sham groups, when the probability value was statistically significant. An LSD t-test was applied for multiple comparisons.

Using Doppler echocardiography, as shown in Fig. 5, peak flow velocity of the aorta (PFVA) was significantly decreased compared to the sham group from four to six weeks in the AAC rats. Additionally, the E wave was significantly decreased in the AAC rats at all time points, beginning as early as three weeks post surgery (Fig. 5B).

**Fig. 6.**
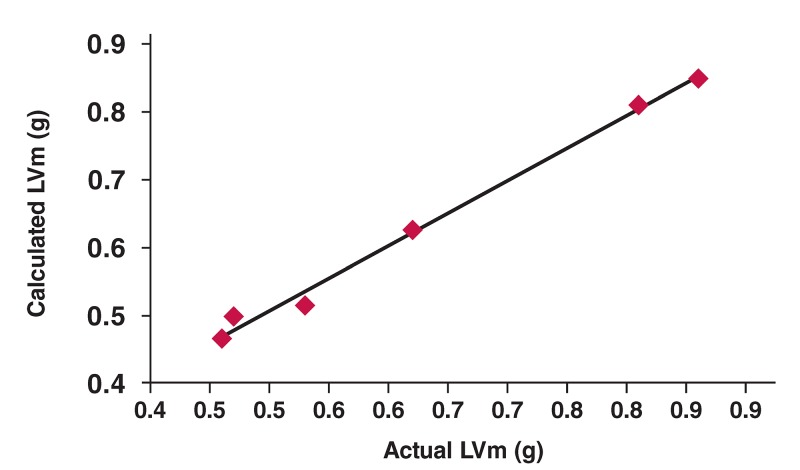
Linear regression analyses of actual heart weights versus LVm values calculated in vivo using echocardiography at all points. Data at each point are the average weights of AAC and sham groups at three, four and six weeks, respectively.

As shown in Table 1, LVMI in the AAC animals was significantly increased as early as three weeks post surgery, and remained elevated compared to the sham controls. The same trend was observed in HMI (*p* < 0.01). By four weeks post AAC, the ratio of LVMI and HMI were markedly increased, reaching 201 and 191% of the sham values, respectively. At all time points, the AAC model caused no significant difference in body weight compared to their respective sham controls. In this pressure overload model, LVm values obtained using echocardiography were consistent with the actual tissue weights, with a correlation coefficient of 0.997 (Table 1, Fig. 6).

**Table 1:** General characteristics of the sham and AAC rats

	*Three weeks*		*Four weeks*		*Six weeks*	
*Parameter*	*Sham (n = 7)*	*AAC (n = 6)*	*Sham (n = 8)*	*AAC (n = 6)*	*Sham (n = 7)*	*AAC (n = 7)*
LV weight (g)	0.47 ± 0.04	0.62 ± 0.08**	0.46 ± 0.04	0.86 ± 0.04**	0.53 ± 0.03	0.81 ± 0.17**
Heart weight (g)	0.58 ± 0.04	0.71 ± 0.09**	0.57 ± 0.05	1.02 ± 0.07**	0.65 ± 0.03	0.95 ± 0.19**
Body weight (g)	189 ± 17	173 ± 13	216 ± 17	201 ± 5	246 ± 17	217 ± 30
LVMI (mg/g)	2.46 ± 0.16	3.55 ± 0.23**	2.13 ± 0.06	4.28 ± 0.13**	2.15 ± 0.10	3.72 ± 0.65**
HMI (mg/g)	3.07 ± 0.16	4.11 ± 0.22**	2.65 ± 0.07	5.07 ± 0.26**	2.65 ± 0.13	4.40 ± 0.71**
LV weight (g)^a^	0.496 ± 0.099	0.625 ± 0.160*	0.464 ± 0.085	0.849 ± 0.081**	0.513 ± 0.100	0.810 ± 0.359**

^a^LV mass was calculated in vivo using echocardiography. *p < 0.05 vs sham control, **p < 0.01 vs sham control.

**Fig. 7.**
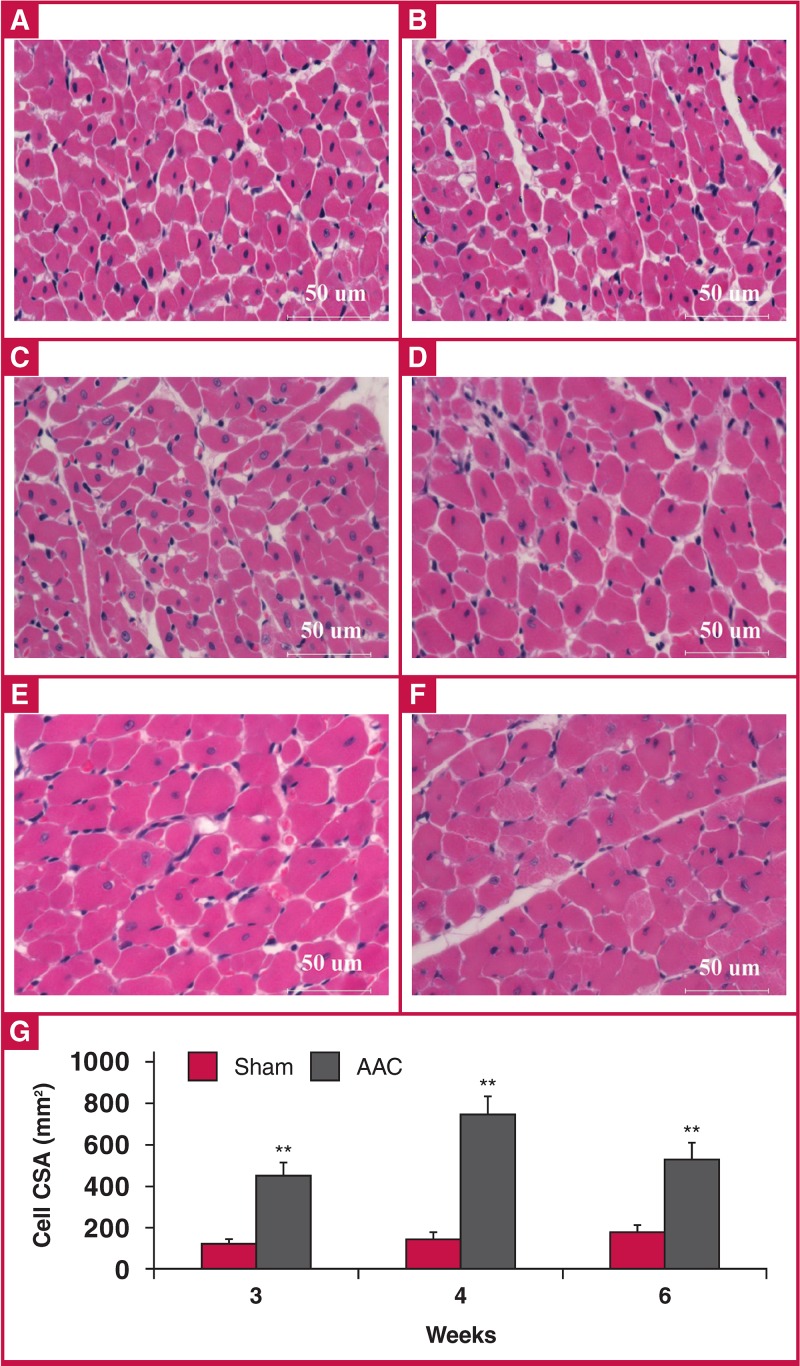
Assessment of cardiomyocyte cross-sectional area (CSA). (A–F) H&E staining (× 400) and myocyte cross-sectional area. Data are presented as mean ± SEM. **p < 0.01 vs sham control. A: sham rats at three weeks, B: sham rats at four weeks, C: sham rats at six weeks, D: AAC rats at three weeks, E: AAC rats at four weeks, F: AAC rats at six weeks, G: comparison of cardiomyocyte CSA between the sham and AAC groups. ANOVA was performed to compare the AAC and sham groups when the probability value was statistically significant. An LSD t-test was applied for multiple comparisons.

Fig. 7 shows morphometric images and data from three, four and six weeks post surgery, comparing myocyte crosssectional area (CSA) between the sham and AAC animals with H&E-stained sections. Pressure overload induced a profound cardiomyocyte hypertrophy in the ACC rats compared with the shams, with a 376% increase in CSA at three weeks, 515% at four weeks, and 294% at six weeks.

**Fig. 8.**
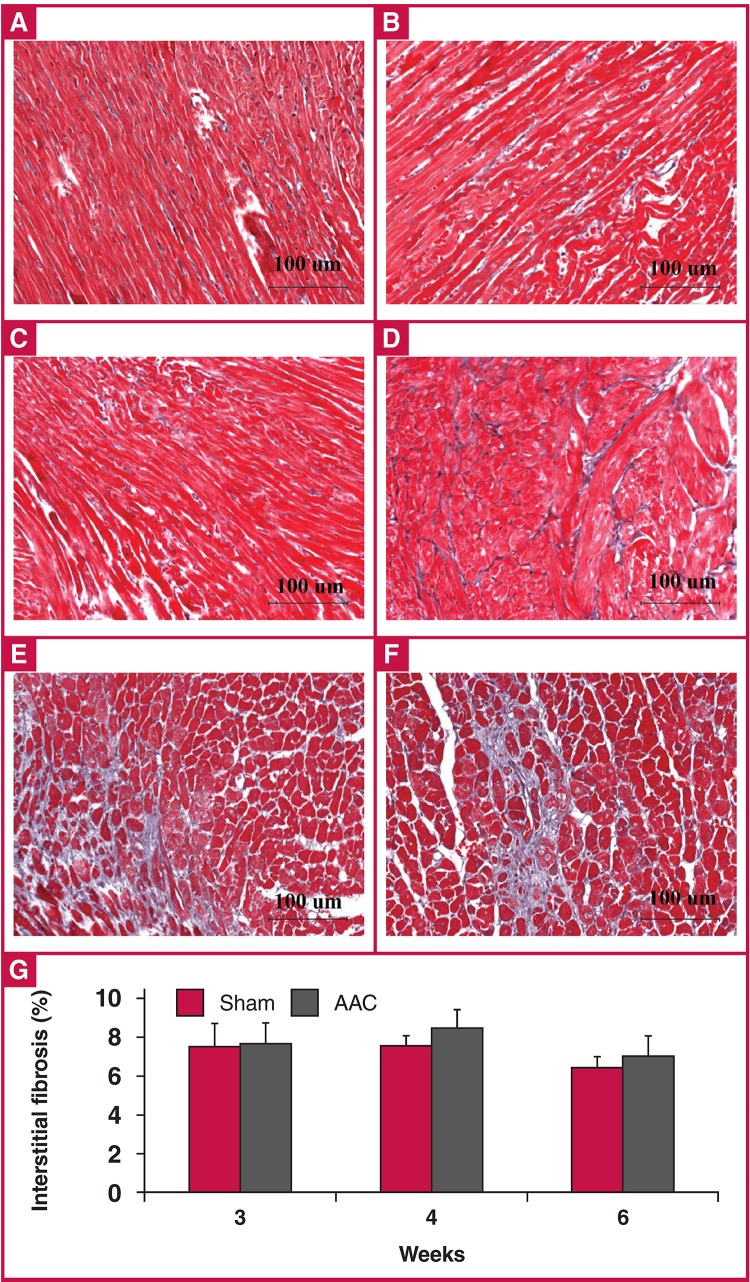
Comparison of interstitial fibrosis between sham and AAC groups using Masson trichrome staining (A–F) (× 200). Data are presented as mean ± SEM. All p-values are > 0.05 vs sham control. A: sham rats at three weeks, B: sham rats at four weeks, C: sham rats at six weeks, D: AAC rats at three weeks, E: AAC rats at four weeks, F: AAC rats at six weeks, G: quantitative analysis of interstitial fibrosis between the sham and AAC groups. ANOVA was performed to compare the AAC and sham groups when the probability value was statistically significant. An LSD t-test was applied for multiple comparisons.

**Fig. 9.**
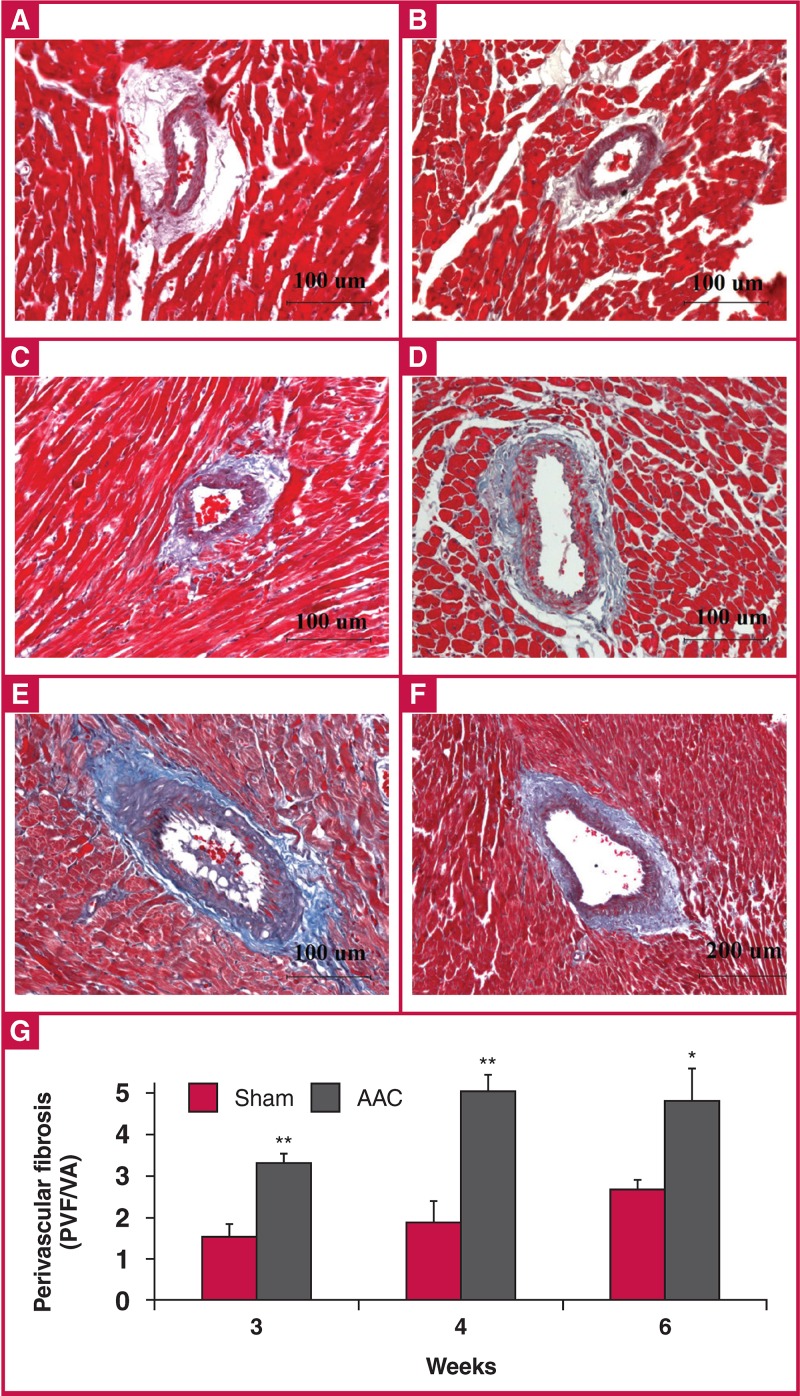
Comparison of perivascular fibrosis between the sham and AAC groups using Masson trichrome staining (A–E) (× 200), (E) (× 100). Data are presented as mean ± SEM. *p < 0.05 vs sham control, **p < 0.01 vs sham control. A: sham rats at three weeks, B: sham rats at four weeks, C: sham rats at six weeks, D: AAC rats at three weeks, E: AAC rats at four weeks, F: AAC rats at six weeks, G: quantitative analysis of perivascular fibrosis between the sham and AAC groups. ANOVA was performed to compare the AAC and sham groups when the probability value was statistically significant. An LSD t-test was applied for multiple comparisons.

Interstitial and perivascular fibrosis measurements are presented in Fig. 8 and Fig. 9. The degree of interstitial fibrosis was elevated in the AAC group compared to the control group at all time points, but did not reach statistical significance (Fig. 8). In the AAC group, perivascular fibrosis was significantly increased at all time points, especially at four weeks (269% of sham values) (Fig. 9).

Plasma BNP levels were significantly increased in a timedependent manner in the AAC group compared to the sham group (*p* < 0.01, Table 2). BNP plasma concentrations in the AAC group at six weeks post AAC surgery were 1.37-fold the level observed at four weeks. However, there were no significant differences in the sham groups between BNP concentrations at four and six weeks post surgery.

**Table 2:** BNP plasma concentration in sham and AAC rats

****	*Three weeks*	*Four weeks*	*Six weeks*
Sham (pg/ml)	117.23 ± 10.49	116.34 ± 8.03	113.72 ± 10.71
AAC (pg/ml)	477.69 ± 22.76**	577.22 ± 24.31**	653.29

**p < 0.01 vs sham control

## Discussion

LV pressure overload can be induced by constricting the ascending aorta, aortic arch or the abdominal aorta in rats, mice or dogs.^13^ AAC is a model of chronic pressure overload that promotes LVH. In previous studies, adult rats weighing 200–300 g^3^,^4^,^14^ were typically used to create the AAC model; however, younger rats weighing 80–100 g were used in our study. The main advantage of using young animals is to produce overload pressure gradually via aortic constriction as the animals age, which is similar to the chronic process of cardiac hypertrophy caused by hypertension.^2^

Our preliminary study confirmed that constricting the abdominal aorta between the branches of the coeliac and anterior mesenteric arteries was more effective than constricting the aorta above the coeliac artery, and was associated with lower mortality rates and no difference in the timing and progression to myocardial hypertrophy. Both methods are better than constricting the abdominal aorta above the left renal artery for the development of myocardial hypertrophy. In the present study, we constricted the abdominal aorta between the branches of the coeliac and anterior mesenteric arteries to a diameter of 0.55 mm, rather than constriction above the left renal artery to a diameter of 0.80 mm, as in most previous studies.^3^-^6^

Pressure-overload LVH has previously been induced using AAC for six weeks.^15^ However, in our experiment, after three weeks, the rats developed significant cardiac hypertrophy with a lower acute (24-hour) mortality rate (8% compared with 15%) than in previous reports.^3^,^5^

Furthermore, the length of the abdominal aorta between the branches of the coeliac and anterior mesenteric arteries is short, therefore the constriction site is circumscribed. Our approach was favourable compared with the traditional method, which defined the constriction site above the left renal artery branch of the abdominal aorta, because there are two major branches here (coeliac and anterior mesenteric arteries), and the constriction site relative to these branches could impact on the progression to myocardial hypertrophy, as describe above. Therefore, development of myocardial hypertrophy in our study was more stable than in traditional methods.

For monitoring changes in hearts subjected to dynamic pressure overload, we used echocardiographic measurements. Echocardiography is a non-invasive method routinely used to investigate changes in cardiac structure and function in various disease states. Echocardiography allows repetitive, non-invasive evaluation in a single live animal, as well as serial determination of cardiac structure and function to follow disease progression and response to therapeutic interventions. This method is far superior and more physiologically relevant than invasive techniques that require open-chest procedures and intubation of vessels, offering no opportunity for time-course studies, since they require time-consuming surgeries, with subsequent euthanasia.

The Sonos 5500 echocardiographic system,^2^-^4^,^7^ a universal type of echocardiography instrument used in clinics, is commonly equipped with a high-frequency transducer that can be used for rodent studies. This study is the first report to use a standard echocardiographic system (IE 33) to assess cardiac structure and function in rats with myocardial hypertrophy. The IE 33 system can clearly show parasternal long-axis and short-axis views, apical four-chamber views of the LV, and the abdominal aorta in rats without requiring an extra transducer. This reduces the cost of investigation in small animals.

Concentric myocardial hypertrophy is a hallmark of chronic pressure overload. Increased ventricular wall thickness induced by overload pressure is initially beneficial to maintain normal cardiac function. However, the heart could convert to heart failure with LV dilatation if the hypertrophic stimulus is maintained. In our study, significant increases in LVPW and IVS with only small decreases in LVIDs were indicative of pure concentric hypertrophy, which is often observed during the early stages of pure pressure overload. Most previous studies have shown late-stage chamber dilatation, signifying the end of the compensatory response and the start of heart failure.^16^ However, in this study, even at six weeks post surgery, there was little change in LVIDs, LVIDd, EDV and ESV, indicating chamber dilatation and dysfunction in ventricular relaxation were not present at this stage of hypertrophy.

FS and EF are commonly used parameters to evaluate systolic function, which is determined by measuring LV end-diastolic and end-systolic diameters with M-mode echocardiography. Since increased afterload may depress stroke volume (SV) in AAC rats, FS and EF were lower in the AAC rats compared to sham rats from three to six weeks. Accordingly, significant decreases in CO were observed in AAC rats, especially at the four-week time point, since CO may be affected by HR and EF (both reduced in the AAC groups).

In this study, we measured PFVA and E waves using echocardiography to evaluate ventricular relaxation and diastolic function. Significant decreases in PFVA and E-wave values in the AAC rats suggested restrictive filling, which may result from the combined effect of elevated ESV and impaired compliance due to wall thickening and/or fibrosis.^17^

Although E/A ratio is a marker for ventricular relaxation, it could not be obtained in this study since the E and A waves were fused in the rats due to their extremely fast heart rates. To distinguish E and A waves, Kokubo *et al.*^7^ used ketamine hydrochloride and xylazine anaesthesia in the animals in order to decrease the HR to 70–80% of normal levels in conscious animals, however control of HR is difficult.

In rats, the heart generally gains ~ 1 g in weight with each 2-mm increase in LV wall thickness. Progressive increases in heart weight, HMI and LVMI, and increased cross-sectional areas of the myocytes (determined using histological analysis) were observed in the ACC rats. Elliott *et al*.^3^ found that echocardiographic determination of LVm is relatively accurate, yet highly overestimated in rats. As LVm increases, a greater degree of LVm overestimation occurs. However, a strong correlation between LVm determined using echocardiography and the actual heart weights of sacrificed rats was observed in the present study, indicating that the parameters measured using echocardiography were closer to the actual values.

Fibrosis is a common response to pressure overload or infarction, to overcome elevated ventricular wall stress. Excessive fibrotic deposits around the small vessels may reduce oxygen and nutrient exchange rates between the blood supply and the surrounding myocardium. Additionally, extensive interstitial fibrosis impedes myocardial relaxation, increasing stiffness in the ventricular wall and reducing LV compliance.

Our data suggest that hypertrophic indices decline from four to six weeks post surgery, with myocardial hypertrophy reaching its peak at four weeks in rats. This observation may be associated with acute pressure overload within four weeks post surgery, and then LV pressure decreases slightly (data not shown), leading to alleviation of myocardial hypertrophy, as previously shown.^18^

As an important biological marker, BNP concentrations in the plasma may increase in line with increased haemodynamic stress, or ventricular cavity expansion in the case of cardiac hypertrophy and heart failure.^14^,^19^-^21^ In the present study, plasma BNP concentrations were associated with the duration of AAC. As the duration extended, BNP levels were progressively elevated, for example, 307, 396 and 474% higher than in the sham groups at three, four and six weeks, respectively.

Some limitations of this study should be considered. E and A waves were not isolated due to the high heart rates of rodents. In addition, the longest time point studied (six weeks) was not long enough to observe the transition from concentric to eccentric hypertrophy. Echocardiographic measurements were performed under anaesthesia, which is known to alter cardiac and respiratory function, even if the doses used were the minimum necessary.

## Conclusion

This study demonstrated that constriction of the abdominal aorta between the branches of the coeliac and anterior mesenteric arteries, to a diameter of 0.55 mm in young rats represents an excellent experimental animal model of pressure overload-induced myocardial hypertrophy. This model induces hypertrophy that occurs at three weeks or earlier, developing to a peak at four weeks, and recovering slightly at six weeks. Changes in cardiac structure and function during the development of cardiac hypertrophy were monitored using a standard ultrasound probe. LVm was determined using echocardiography and was consistent with the actual tissue weights. By using this animal model, we aim to provide a theoretical and experimental foundation for the application of novel drugs, for example, highspecificity and high-affinity receptor antagonists or agonists to intervene in the pathogenesis of clinical LVH.
